# Deciphering a unique biotin scavenging pathway with redundant genes in the probiotic bacterium *Lactococcus lactis*

**DOI:** 10.1038/srep25680

**Published:** 2016-05-10

**Authors:** Huimin Zhang, Qingjing Wang, Derek J. Fisher, Mingzhu Cai, Vandana Chakravartty, Huiyan Ye, Ping Li, Jose O. Solbiati, Youjun Feng

**Affiliations:** 1 Department of Medical Microbiology and Parasitology, Zhejiang University School of Medicine, Hangzhou, Zhejiang 310058, PR China; 2 Department of Microbiology, Southern Illinois University, Carbondale, IL 62901, USA; 3 Department of Microbiology, University of Illinois, Urbana, IL 61801, USA; 4 Institute for Genomic Biology, University of Illinois, Urbana, Illinois 61801, USA

## Abstract

Biotin protein ligase (BPL) is widespread in the three domains of the life. The paradigm BPL is the *Escherichia coli* BirA protein, which also functions as a repressor for the biotin biosynthesis pathway. Here we report that *Lactococcus lactis* possesses two different orthologues of *birA* (*birA*1__LL_ and *birA*2__LL_). Unlike the scenario in *E. coli, L. lactis* appears to be auxotrophic for biotin in that it lacks a full biotin biosynthesis pathway. In contrast, it retains two biotin transporter-encoding genes (*bioY*1__LL_ and *bioY*2__LL_), suggesting the use of a scavenging strategy to obtain biotin from the environment. The *in vivo* function of the two *L. lactis birA* genes was judged by their abilities to complement the conditional lethal *E. coli birA* mutant. Thin-layer chromatography and mass spectroscopy assays demonstrated that these two recombinant BirA proteins catalyze the biotinylation reaction of the acceptor biotin carboxyl carrier protein (BCCP), through the expected biotinoyl-AMP intermediate. Gel shift assays were used to characterize *bioY*1__LL_ and BirA1__LL_. We also determined the ability to uptake ^3^H-biotin by *L. lactis.* Taken together, our results deciphered a unique biotin scavenging pathway with redundant genes present in the probiotic bacterium *L. lactis*.

Biotin (vitamin H) is essential in the three domains of life. Biotin is a covalently-bound enzyme cofactor in central metabolism like the acetyl-CoA carboxylase (ACC) reaction required to form the fatty acid building block, malonyl-CoA^1^. Most bacteria synthesize biotin. However, some bacteria must scavenge biotin from the environment. BioY is a biotin transporter found in many bacteria^2^,^3^,^4^. Generally, BioY is considered to be the substrate-binding component (S component) of the Energy Coupling Factor transport family (ECF), an ATP-binding cassette transporter involved in the uptake of multiple micro-nutrients^5^,^6^. *E. coli* can either synthesize biotin or transport biotin using a non-BioY/ECF mechanism^7^. Interestingly, acquisition of biotin in *Streptococcus suis*, an animal pathogen, is solely dependent on the presence of the BioY transporter because it lacks a biotin synthesis pathway^4^. Hebbeln and coworkers reported the *in vitro* biochemistry of the tripartite biotin transporter BioMNY from the α-proteobacterium *Rhodobacter*^2^. Although the crystal structure of the BioY membrane protein of *Lactococcus lactis* was reported^8^, the regulated expression of *bioY* and biotin sensing mediated by the BirA gatekeeper protein remained unknown.

*L. lactis* IL1403 is a member of a group of low GC Gram-positive bacteria^9^. Generally, it is believed to stay dormant on plants, but grows within the gastrointestinal tract. Given the fact that *L. lactis* vigorously ferments lactose in milk, with production of ATP and lactic acid, *L. lactis* has been widely applied in the dairy industry, such as buttermilk and cheese production^10^. Given its long role in food fermentation, *L. lactis* has been generally recognized as safe (GRAS) status. The presence of *L. lactis* in the intestine of animals and humans significantly benefits the immune system and it is generally regarded as a probiotic bacterium^11^. It seemed unusual that *L. lactis* IL1403 with a small genome (2.37 Mb), which is estimated to be only half of the *E. coli* genome (4.64 Mb for MG1655)^9^, encodes numerous redundant (and/or duplicated) loci in the context of lipid metabolism as well as within the biotin utilization pathway. While unexpected, such an arrangement is not without precedent as a similar scenario was observed in *Paracoccus*^12^. This rare situation where redundant genes of biotin metabolism are present within the minimal genome of *L. lactis* might render some unknown physiological advantage, raising a possibility that it represents a relic of bacterial evolution and selection by its environmental niche^13^,^14^.

In this paper, we performed systematic functional analyses of the redundant genes in the context of biotin metabolism. We demonstrate *in vivo* evidence for biotin transport by *L. lactis* and also determined distinct functional assignments for the two BirA orthologues. Finally, we formulated a working model for biotin scavenging by *L. lactis* (Fig. 1). The atypical occurrence of two different biotin protein ligases might suggest its unique evolution history in adaption to its growing environment and/or ecological niche.

## Results and Discussion

### Redundancy of biotin metabolism genes in *L. lactis*

**Figure 1:**
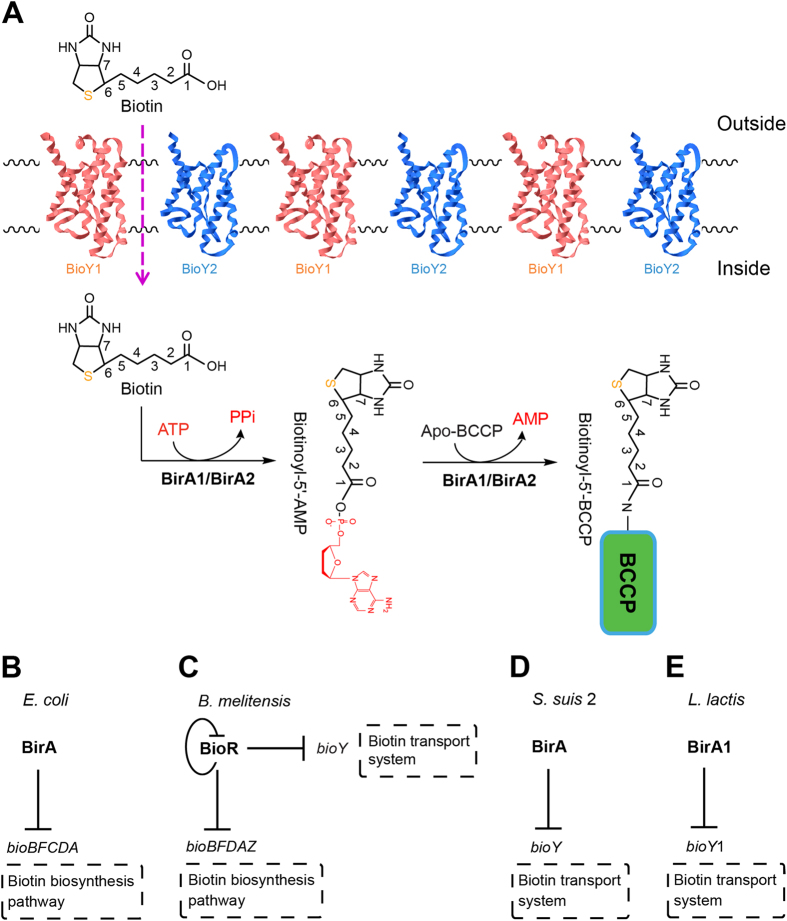
Current models for biotin uptake and BirA biotin protein ligase/repressor functions. (**A**) Proposed biotin uptake and utilization pathway in *L. lactis.* The two half-reactions of BirA-mediated BCCP biotinylation are shown. (**B)** *E. coli* BirA represses the biotin synthesis pathway (**C**). *B. melitensis* BioR represses expression of the biotin synthesis pathway and the biotin transport system (**D)**. Transcriptional repression of the *bioY* gene by the BirA of *S. suis* 2 (**E**). BirA1 represses expression of the *bioY* gene in *L. lactis.*

**Figure 2:**
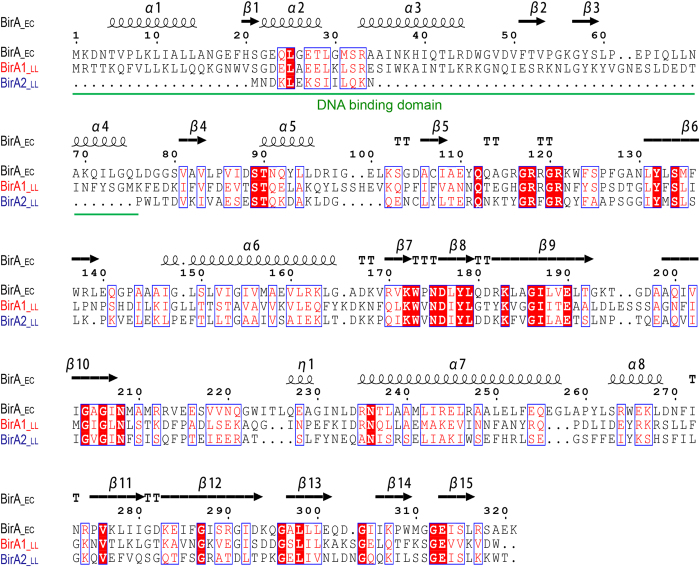
Sequence alignment of the *L. lactis* BirA homologues with the *E. coli* BirA. The multiple alignment was performed using ClustalW2 (http://www.ebi.ac.uk/Tools/clustalw2/index.html), and the final output was processed using the program ESPript 2.2 (http://espript.ibcp.fr/ESPript/cgi-bin/ESPript.cgi). The well-studied BirA_ec protein was used from *E. coli* K-12 MG1655 (Accession no. AAC43075), and the two BirA homologues were derived from *L. lactis* IL1403. BirA1__LL_ (Accession no. NP_267937) is shown in red and BirA2__LL_ (Accession no. NP_268060) is indicated in blue. Identical residues are in white letters with red background, similar residues are in red letters with white background, varied residues are in black letters, and dots represent gaps. The predicted secondary structure of BirA is shown at the top of the alignment. α: α-helix; β: β-sheet; T: β-turns/coils. Designations: EC, *E. coli*; LL, *L. lactis.*

Similar to its closely-related cousin, the zoonotic pathogen *Streptococcus suis*^15^, *L. lactis* is also a low GC Gram-positive bacterium with a reduced genome (35.3% GC percentage, 2.37 Mb)^9^. However, it seemed unusual that the genetic organization of *L. lactis* features gene duplications and/or redundancy in the context of biotin metabolism (Table S1). Unlike *S. suis* that encodes only one *birA* gene and one *bioY* gene, *L. lactis* has two *birA* orthologues (called *birA*1 [L0191] and *birA*2 [L0192], Table S1) and two *bioY* orthologues (*bioY*1 [L24031] and *bioY*2 [L1011], respectively (Table S1). The *birA*2 gene encodes a putative “simple” biotin protein ligase (BPL) that lacks the N-terminal DNA binding motif found in the putative dual-functional *birA*1 (Figs 1 and 2). While the presence of multiple copies of *birA* and *bioY* homologues is atypical, two copies of *birA* have been found in *Francisella*^16^ and two different *bioY* orthologues are present in *Paracoccus*^12^. Apart from redundancies in *birA* and *bioY*, redundancy is also found within the fatty acid synthesis loci in *L. lactis* (Table S1). Three loci of *fabG* (3-oxoacyl-ACP reductase) exist in *L. lactis*, namely *fabG*1 (L0185), *fabG*2 (L27694), and *fabG*3 (L1530) (Table S1). Of note, Wang and Cronan reported that the *fabG*2 is not an active 3-oxoacyl-ACP reductase^17^, indicating a possible role of these *fabG* loci in sugar metabolism. Moreover, *L. lactis* has two loci (*fabZ* [L0188] and *fabZ*1 [L160425]) that encode a 3-hydroxyacyl-ACP dehydratase (Table S1). Two fatty acid degradation (*fad*) genes (*fadA* [L25946] *fadD* [L54546]) also are present in *L. lactis*, whereas the fad system is cryptic for *Streptococcus* (Table S1). These gene arrangements suggest an unexpected complexity of lipid metabolism (including biotin utilization) in *L. lactis* compared with its close relative, *S. suis* (Table S1).

### Characterization of the Two BirA homologues

BPL has been classified into two groups: the Group I BPLs lack an N-terminal DNA-binding motif whereas the Group II BPLs (BirAs) have an N-terminal DNA-binding domain^4^. The paradigm Group II BPL is the *E. coli* BirA and such enzymes also are found in the Archaea, suggesting that the group II version may be the ancestor of the BPL enzyme family. Based upon annotation, *L. lactis* IL1403 likely possesses two types of BPLs, which are referred to here on as BirA1__LL_ (323 residues, a putative Group II BPL) and BirA2__LL_ (250 aa, a putative Group I BPL). Multiple sequence alignments of the two BirA proteins showed that they exhibit 20.7% identity and 34.6% similarity compared to the *E. coli* BirA (Fig. 2).

**Figure 3:**
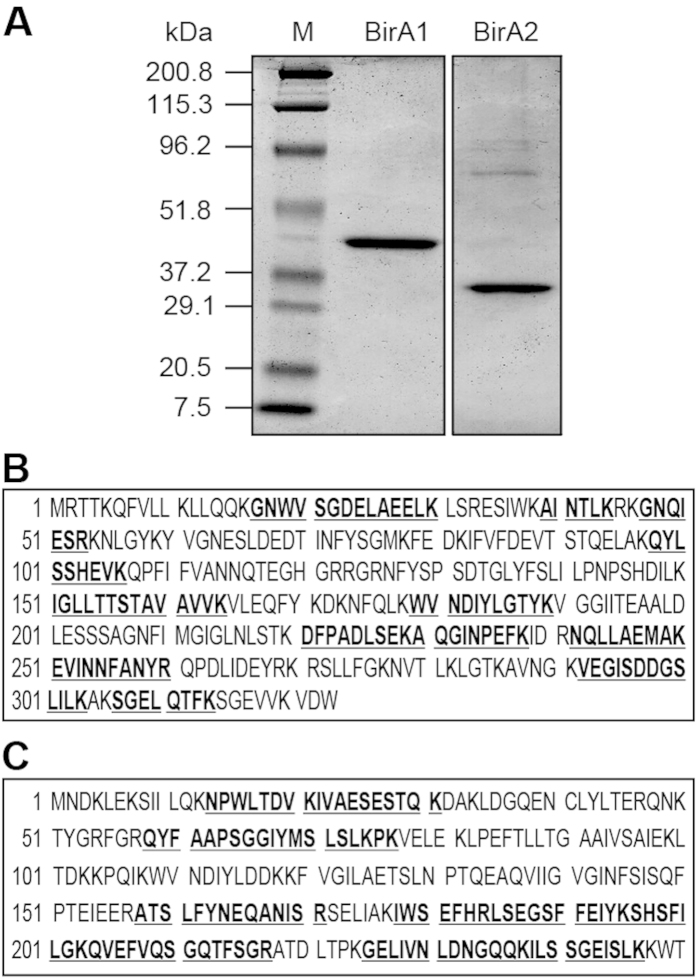
Purification and MS identification of *L. lactis* BirA1 and BirA2. (**A)** 12% SDS-PAGE profile of the two purified BirA proteins (BirA1 and BirA2). A pre-stained protein standard marker (M) was used in the first lane and marker sizes are indicated in kDa to the left of the image. Results for liquid chromatography quadrupole time-of-flight MS verification of the two recombinant proteins, BirA1 (in Panel **B**) and BirA2 (in Panel **C**). The peptide fragments that matched the database sequence are indicated in bold and underlined type. The coverage was 36.8% for BirA1, and 45.6% for BirA2, respectively.

To test the BPL activity of the two *L. lactis* IL1403 BirA proteins, we purified the hexahistidine-tagged proteins to homogeneity. SDS-PAGE profiles of the purified recombinant proteins suggested that BirA1__LL_ is around 40 kDa, whereas the BirA2__LL_ is estimated to be about 30 kDa (Fig. 3A). To further verify their identity, MALDI-TOF was used to analyze digested polypeptide fragments. The MS results supported that the two purified, recombinant proteins are BirA1__LL_ (36.8% coverage) and BirA2__LL_ (45.6% coverage) (Fig. 3B,C).

**Figure 4:**
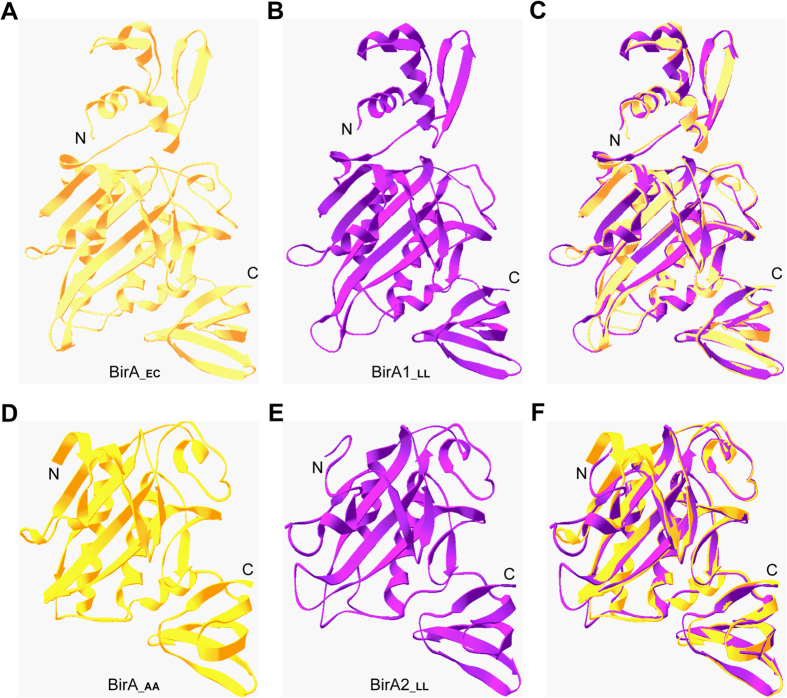
Structural analyses of two BirA homologue proteins from *L. lactis.* The *E. coli* BirA structure (in Panel **A**) and the modeled structure of *L. lactis* BirA1 (BirA1__LL_) (in Panel **B**) exhibit strong structural similarity. (**C)** Structural superposition of BirA1__LL_ with BirA__EC._ The BirA__EC_ structure (PDB: 1HXD) is in yellow, whereas that of BirA1__LL_ (with 28.6% identity to BirA__EC_ structure) is indicated in purple. An overall view of *Aquifex aeolicus* BirA protein (BirA__AA_) structure (in Panel **D**) and the modeled structure of BirA2__LL_ protein (in Panel **E**) (**F)**. Structure superposition of BirA2__LL_ with BirA__AA._ The structure of BirA__AA_ (PDB: 3EFS) is in gold, whereas the modeled structure of BirA2__LL_ using BirA__AA_ as the template with 32.4% identity is highlighted in purple. Designations: AA, *Aquifex aeolicus*; LL, *L. lactis*; N, N-terminus; C, C-terminus.

To better visualize the architectures of the two distinct forms of *L. lactis* BPLs, structural modelling was carried out. In similarity to that of the *E. coli* BirA (Fig. 4A)^18^, BirA1__LL_ also possesses the N-terminal DNA-binding motif and the C-terminal enzymatic domain (Fig. 4B,C), fitting the criteria for the Group II BPLs. The structure of the BirA2__LL_ was almost identical to that of the *Aquifex aeolicus* BirA, a member of Group I BPLs^19^ (Fig. 4D–F), supporting its classification as a Group I BPL.

### Comparing the BPL activity of the two *L. lactis* BirA proteins

**Figure 5:**
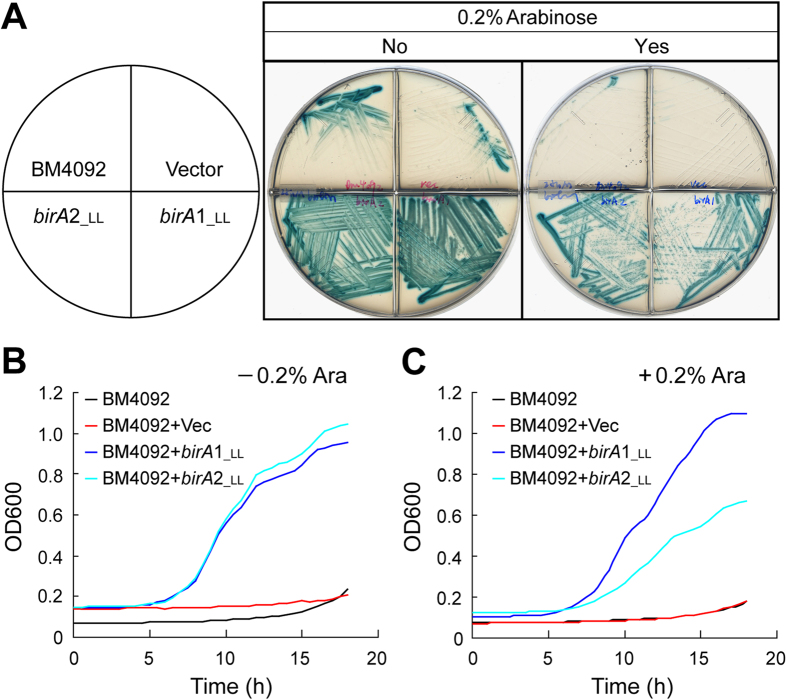
Both *L. lactis birA* homologues (LL*birA*1__LL_ and LL*birA*2__LL_) can complement the *E. coli birA* Km mutant. (**A**) *L. lactis birA* genes (*birA*1_*_LL*_ and *birA*2__LL_) can support growth of the *E. coli birA1* Km mutant on minimal media supplemented with 25 nM biotin. BM4092 is an *E. coli birA*1 Km mutant strain carrying a *bio-lacZ* fusion. 0.5 μM X-gal was added into the media to monitor transcription of the *bio-lacZ* by BirA. Yes or No indicates whether the agar plate contains 0.2% arabinose. The inoculated plates were grown at 30 °C for ~20 hours. Representative plate results are shown. Designations: Ara, arabinose; Vec, the pBAD24 empty vector, LL, *L. lactis*. Growth curves of the BM4092 *birA* Km mutant complemented with either plasmid-borne *L. lactis birA* genes (either *birA*1_*_LL*_ or *birA*2__LL_) in liquid minimal media with (**B**) or without (**C**) the addition of 0.2% arabinose. 25 nM of biotin was supplemented into the M9 liquid media. Bacterial growth was measured by optical density at 600 nm, which is automatically recorded using a *BioScreen C* instrument. Each growth curve assay was carried out in triplicate and the average is shown in this plot^32^.

BPL activity was measured using the well-studied *E. coli* model together with *in vitro* enzymatic assays. The model *E. coli* strain for functional complementation of *birA* homologues is the *birA*1 mutant BM4092, which lacks the ability to synthesize biotin or to transport biotin with high affinity^20^. As expected, the BM4092 strain either with or without the empty vector pBAD24 did not grow on M9 agar plates supplemented with 25 nM of biotin (Fig. 5A). In contrast, the arabinose-induced expression (and even basal expression) of both *birA*1__LL_ and *birA*2__LL_ supported the growth of BM4092 under the non-permissive biotin levels (25 nM) (Fig. 5A). The bacterial growth curves of the BM4092 derivatives in liquid media also gave similar results to those obtained from the agar plates (Fig. 5B,C). Of note, overexpression of *birA*1__LL_ seemed slightly toxic for *E. coli* in that bacterial growth of the BM4092 strain supplemented with 0.2% arabinose was significantly inhibited when compared with growth conditions lacking the inducer, arabinose (Fig. 5C). Together, these data provided *in vivo* evidence that the two BirA orthologues of *L. lactis* (BirA1__LL_ and BirA2__LL_) have BPL activity.

**Figure 6:**
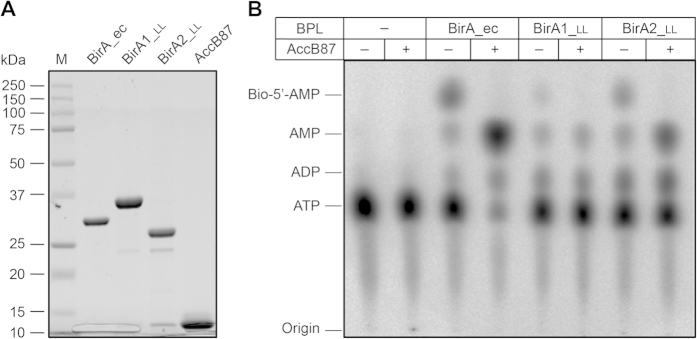
Assessment of BPL activity for the two BirA homologues (BirA1__LL_ and BirA2__LL_) using TLC. (**A**) SDS-PAGE profile of the affinity-purified BPL proteins and the AccB87substrate (**B)**. TLC-based analyses of BPL activity. Minus means no addition of either BPL protein or AccB87 substrate protein and plus denotes addition of AccB87. Products of the BPL reaction were separated on TLC and visualized using phosphor-imaging. Abbreviations: M, protein marker (Bio-Rad); ec, *E. coli*; LL, *L. lactis*; BPL, biotin protein ligase.

Subsequently, BPL function of the BirA orthologues was further compared using *in vitro* assays to assess biotinylation activity. In this reaction, three versions of BirA were included (BirA_ec, BirA1__LL_ and BirA2__LL_), and the domain of the AccB protein targeted for biotinylation by BPLs (designated as AccB87 or BCCP87) was used as the substrate (Fig. 6A). In principle, the conserved lysine at position 122 (K122) of AccB87 should be biotinylated upon the presence of a functional BPL (Fig. 1A). The conversion of α-^32^P-labeled ATP and biotin to the intermediate product, biotinoyl-AMP, by the BPLs (Fig. 1A) was directly visualized using thin layer chromatography (TLC) (Fig. 6B). The TLC approach also provided indirect proof of the second ligase partial reaction that occurs upon introduction of the acceptor protein AccB87 (*i.e.*, transferring of biotin from biotinoyl-5′-AMP to the AccB87 lysine) resulting in loss of the biotinoyl-5′-AMP intermediate and formation of AMP (Figs 1A and 6B). The increased consumption of ATP in the presence of AccB87 (most notably for BirA-ec) was due to the fact that in absence of the acceptor protein, biotinoyl-AMP remains tightly bound within the ligase active site so that only one molecule of biotinoyl-AMP was formed per molecule of ligase (*i.e*., biotinoyl-AMP synthesis is not catalytic). Biotin transfer to an acceptor protein allows for catalysis and continued conversion of ATP to biotinoyl-AMP. These results further confirmed BPL activity.

**Figure 7:**
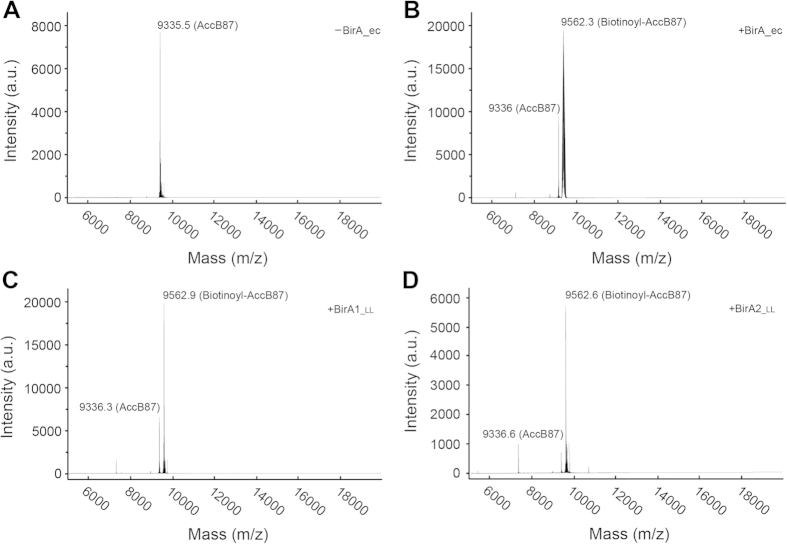
MS-based identification of AccB87 biotinylation by *L. lactis* BirA1/BirA2. (**A)** MALDI-TOF determination of the molecular weight of the un-biotinylated AccB87 polypeptide. The calculated mass for AccB87 is 9335.5~9336.6 and the expected mass for biotinoyl-AccB87 is 9562.3~9562.9. (**B)** The formation of the biotinylated AccB87 by *E. coli* BirA. (**C**) Biotinylation of AccB87 by *L. lactis* BirA1. (**D)** Biotinylation of AccB87 by *L. lactis* BirA2. Designations: minus denotes no addition of BirA enzyme, plus denotes addition of the BirA protein. Abbreviations: ec, *E. coli*; LL, *L. lactis*.

Our results demonstrate that both BirA proteins from *L. lactis* possess the ability to convert biotin and [α-^32^P]-ATP to the canonical biotinoyl-5′-AMP intermediate (Fig. 6B) and indirectly support transfer of the biotin moiety to the AccB87 acceptor protein (Fig. 6B). The most striking difference between BirA1__LL_ and BirA2__LL_ was that upon addition of the acceptor protein, the ratio of AMP to ATP in the BirA2__LL_ assay is appreciable higher than that seen in the BirA1__LL_ assay and BirA2__LL_ appears to form more of the biotinoyl-5′-AMP intermediate (Fig. 6B). This indicates that BirA2__LL_ catalyzes biotin attachment more quickly than BirA1__LL_. The *E. coli* BirA consumed the majority of the α-^32^P-labeled ATP whereas no appreciable increase in ATP consumption was seen in the *L. lactis* BirA assays (Fig. 6B). This implies that the BirA_ec is the most active version amongst the three BPL enzymes under the conditions tested. Mass alteration of the AccB87 caused by biotinylation was directly measured using MALDI-TOF as previously described. In our assays, the mass for AccB87 was calculated to be 9335.5~9336.6 (Fig. 7A), and the mass for the biotinoyl-AccB87 was determined to be 9562.3~9562.9 (Fig. 7B–D). Collectively, the data illustrated that: 1) the two versions of *L. lactis* BirA (BirA1__LL_ and BirA2__LL_) functioned as BPLs with weak activity; 2) BirA2__LL_ was more catalytically active relative to BirA1__LL_.

### Binding of *L. lactis* BirA to the *bioY* genes

**Figure 8:**
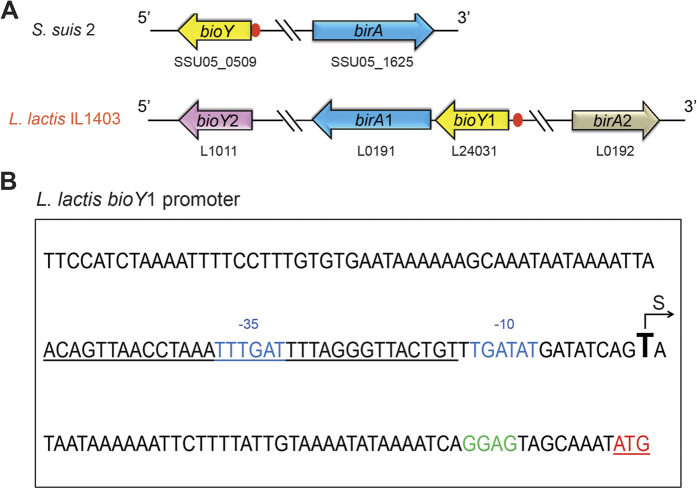
Genetic organization of the *birA* and *bioY* loci and the *L. lactis bioY*1 promoter. (**A**) Genetic organization of *birA* and *bioY* loci. The *birA* (referred to *birA*1) and *bioY* (referred to *bioY*1) loci are highlighted in blue and yellow, respectively. The red circle denotes the predicted BirA-binding site. The *birA*2 and *bioY*2 loci are indicated with silver and purple arrows. (**B)** The *L. lactis bioY* promoter “S” denotes the putative transcriptional start site and the red “ATG” is the translation initiation site. The letters “GGAG” in green refer to the ribosome binding site. The “−35” and “−10” regions are indicated in blue. The predicted BirA-binding site is underlined in black.

Relative to the scenario seen in the closely-relative cousin, *S. suis*, the genomic context of the *bio* loci is complex due to the presence of duplicated genes for *birA* and *bioY* (Fig. 8A). The putative BirA1 DNA binding site (ACA GTT AAC CTA AAT TTG ATT TTA GGG TTA CTG T) was detected in front of the *bioY*1 promoter region (Fig. 8B). According to the position of the transcriptional start site “T” predicted with the Neutral Network Program of Promoter Prediction (http://www.fruitfly.org/seq_tools/promoter.html), the BirA1-binding site appears to overlap the “−10” to “−35” promoter regions (Fig. 8B), suggesting the transcription of *bioY*1 might be negatively regulated by BirA1 in *L. lactis*.

**Figure 9:**
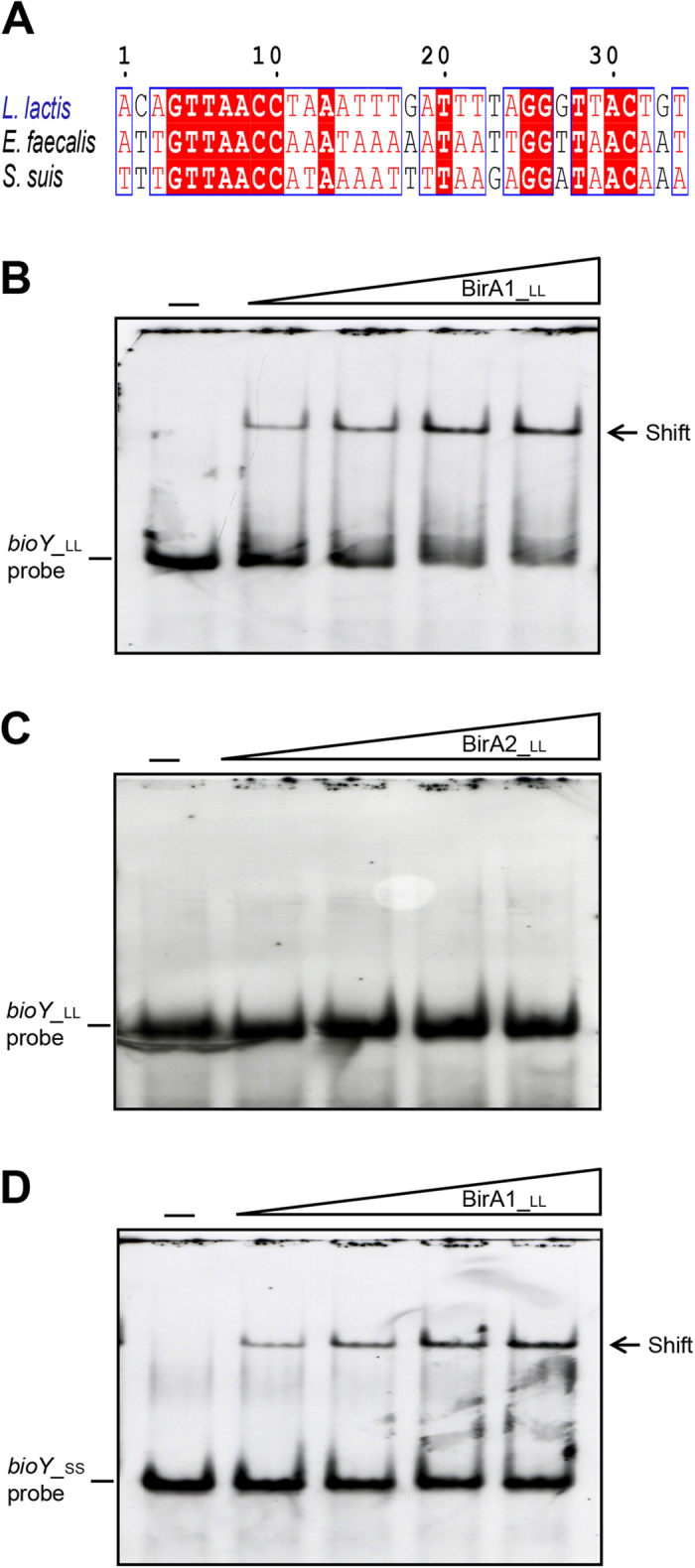
Binding of *L. lactis* BirA1 to the *bioY* promoter region. (**A**) Multiple sequence alignment of the predicted BirA-recognized palindromes localized in the *bioY* promoter regions Identical residues are in white letters with red background, similar residues are in red letters with white background, whereas non-conserved residues are in grey letters. Electrophoretic mobility shift assay (EMSA) of the binding of *L. lactis* BirA1 to the promoter regions of *bioY* from *L. lactis* (in Panel **B**) and the closely-related organism *Streptococcus suis* (in Panel **D**). (**C**) EMSA-based evidence that *L. lactis* BirA2 protein cannot bind to the promoter region of *L. lactis bioY.* The minus sign denotes no addition of either BirA1__LL_ or BirA2__LL_ protein, and the DIG-labeled probe shifted by BirA1__LL_ protein is indicated with an arrow. The concentrations of BirA1__LL_ (BirA2__LL_) in the right four lanes of each panel were (from left to right) 3, 6, 9 and 12 pmol, respectively. The protein samples were incubated with 0.2 pmol of DIG-labeled probe in a total volume of 15 μl. To make biotinoyl-AMP, the ligand of BirA protein, 100 uM biotin plus 100 uM ATP was added into the gel shift system and incubated for ~1 hour. Designations LL and SS denote *L. lactis* and *S. suis*, respectively. BirA1__LL_ and BirA2__LL_ denote the two putative versions of *L. lactis* BirA protein, whereas *bioY*__LL_ and *bioY*__SS_ denote the *bioY* gene from *L. lactis* and *S. suis*, respectively. A representative graph from three independent gel shift assays is presented here. 7.5% native PAGE gels were used for the assays.

To test the function of the predicted BirA site (Figs 8A and 9A), electrophoresis mobility shift assays (EMSA) were conducted using the purified BirA1__LL_ and BirA2__LL_ (Fig. 9B–D). EMSAs confirmed that BirA1__LL_ effectively binds the *bioY1* probe in a dose-dependent manner (Fig. 9B), whereas BirA2__LL_, which lacks the putative N-terminal DNA binding motif, did not bind to the probe (Figs 2 and 9C). Interestingly, the *S. suis bioY* (*bioY*__SS_) promoter has the ability to interact with the *L. lactis* BirA1__LL_ (Fig. 9D). A similar scenario was found in the case of *S. suis* BirA in that it also binds the *L. lactis bioY* promoter. Therefore, we anticipated that crosstalk would be present between the *bioY* and BirA of both *S. suis* and *L. lactis*. Given the fact that interaction occurs between the BirA1 and *bioY*1 promoter, while *bioY2* lacks a predicted BirA1 binding site, it might be of interest to probe the physiological relevance of this regulation mechanism to biotin assimilation.

### The ability of *L. lactis* to transport ^3^H-biotin

**Figure 10:**
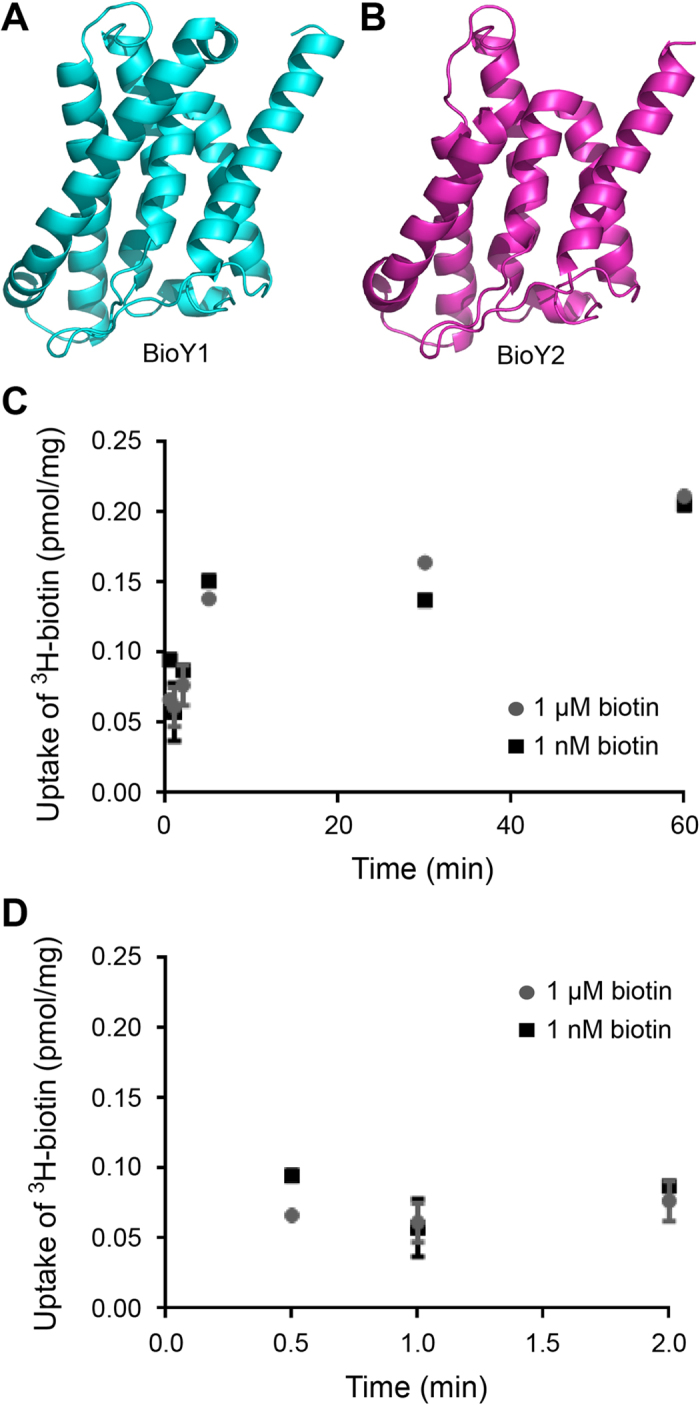
Structural and functional analysis of the *L. lactis* IL1403 biotin transporters. (**A**,**B**) Modelled structures of the BioY1 and BioY2 biotin transporter proteins of *L. lactis* IL1403. Using the S component structure (PDB: 4DVE) from a BioY ECF-type ABC transporter^8^ as the structural template, structural modeling of BioY1 and BioY2 was performed with the Swiss-Model program and the ribbon structure was produced with the software PyMol (**C**) Time trial analysis of transport of ^3^H-biotin by *L. lactis* grown under biotin replete (1 μM) or deplete (1 nM) conditions (**D**) Expanded curve of ^3^H-biotin transport by *L. lactis* at early time points. Bacteria were grown in the presence of either 1 nM or 1 μM biotin, pelleted and washed to remove external biotin, and then suspended in PBS plus 0.5% glucose for transport assays. For transport assays, samples were incubated with 0.5 μM ^3^H-biotin at 30 °C for 0.5, 1, 2, 5, 30, or 60 minutes. Reactions were halted via dilution in ice cold PBS and bacteria were collected on 0.45 μm filters. Filters were then mixed with scintillation fluid and DPM were counted using a Packard Scintillation counter. Average results for the time trials are shown in Panel **C** (time points less than 2 minutes are shown in Panel **D**).

Structural modelling against a solved BioY structure^8^ suggested that both BioY1__LL_ (Fig. 10A) and BioY2__LL_ (Fig. 10B) consists of seven α-helices and exhibits appreciable similarity in their overall configuration. Given the fact that the *L. lactis* encoded two BioY transporters, we hypothesized that it would be capable of robust biotin transport. *L. lactis* cultures were grown in the presence of either limiting biotin or excess biotin (either 1 nM or 1 μM) for 4.5 hours, pelleted and washed with PBS to remove free biotin, and used for ^3^H-biotin transport assays (Fig. 10C,D). Relative transport of ^3^H-biotin by biotin depleted cultures (predicted to produce both BioY transporters) and biotin replete cultures (predicted to require single transporter) was similar over the intervals measured. Minor differences between the samples were only observed at the initial transport time point (Fig. 10D). In contrast, at one minute and later, uptake/accumulation of biotin is roughly equal (Fig. 10C). It seems likely that the increment of exogenous biotin tested did not significantly augment activity of biotin uptake.

## Conclusions

The data reported here suggests a specific pathway for biotin utilization in the probiotic bacterium *L. lactis* (Fig. 1) differing with the closely related animal pathogen *S. suis* in the redundancy (and/or duplication events) of the *birA* and *bioY* loci (Fig. 8). *L. lactis* is anticipated to be a biotin auxotroph in that it lacks a full biotin biosynthesis pathway. Unlike the regulatory machinery for biotin uptake in *S. suis* in which BirA modulates the transcription of the *bioY* gene, *L. lactis* has two BioY transporters responsible for biotin uptake, and two BPL enzymes (BirA1__LL_ and BirA2__LL_) catalyzing protein biotinylation. Among the two *bioY* genes (*bioY*1__LL_ and *bioY* 2__LL_), it seems likely that transcription of *bioY*1__LL_ is regulated by BirA1__LL_, whereas the expression of *bioY*2__LL_ is constitutive (Fig. 1). This unique mechanism might guarantee *L. lactis* possesses the ability to respond to fluctuating levels of biotin in the environment and/or the gastrointestinal tract.

To the best of our knowledge, regulation of bacterial biotin metabolism occurs by at least three diverse mechanisms represented by the *E. coli* BirA^4^, *Agrobacterium* BioR^20^, and *Mycobacterium smegmatis* BioQ^21^. The regulated transport of biotin is exemplified by BioR of *Brucella*^22^ plus *Paracoccus*^12^ and *S. suis* via BirA. The atypical occurrence of two distinct, functional biotin protein ligases in *L. lactis* adds to the breadth of unique examples for bacterial biotin utilization (Fig. 1). In the organism *Francisella*, we also noted the atypical presence of two different BirA orthologues^16^. However, we failed to detect any orthologue of the BioY transporter^16^, ruling out the possibility of regulated transport of biotin. Unlike the scenario with *Francisella* where the BirA bifunctional protein can cross-talk with the *E. coli bio* operon *in vitro* and *in vivo*, the *L. lactis* BirA1 cannot regulate expression of the *E. coli bio* operon (note: blue color is due to appreciable expression of the *bio-lacZ* fusion, Fig. 5). Of note, the *L. lactis* BirA can crosstalk with the *S. suis bioY* gene (Fig. 9D), and *vice versa*. Consistent with the scenario in *E. coli*, the *accB* gene (L0187) encoding the biotin-acceptor protein was detected in the genome of *L. lactis* (Table S1). The reduced BPL activities of the two individual BirA homologues from *L. lactis* compared to the BPL activity of the single *E. coli* BirA (Fig. 6) might argue that redundancy of BirA_LL_ is required to fulfill the need for protein biotinylation in *L. lactis*. The inability of *L. lactis* to synthesize biotin and the unique ecosystem of the gastrointestinal tract which is filled with a variety of nutritional elements and competing bacteria could select for the redundancy in biotin scavenging pathways or conversely, the gene organization might simply be a relic of bacterial evolution. Future work could explore the regulation of transporter production under different biotin conditions as well as differences in transporter efficiency.

## Methods

### Bacterial strains and growth conditions

The *E. coli* strains used included MG1655, BM4092 (Km mutant of *birA*)^20^, DH5α, and BL21 (DE3). The strains of *L. lactis* IL1403^9^ and *S. suis* 2^15^ were used for functional analyses. Luria Bertani (LB) and rich broth (RB) were used for the growth of *E. coli* and both Todd Hewitt Broth (THB) and minimal medium were used for the maintenance of *L. lactis* IL1403 and *S. suis* 2^23^. When necessary, antibiotics were added as follows (in mg/liter): sodium ampicillin, 100; and kanamycin sulfate, 50.

### Plasmids and genetic manipulations

The two *birA* genes (*birA*1 [L0191] and *birA*2 [L0192]) were amplified by PCR and cloned into the expression vector pET28(a) to create histidine-tagged proteins for affinity purification and the arabinose-inducible vector pBAD24 for gene complementation experiments as previously described^24^. The derivatives of pET28 were introduced into BL21 (DE3) for protein production, whereas the pBAD24 derivatives expressing *birA* were transformed into the Km mutant of *birA* (BM4092) for functional assay of *birA*^25^,^26^. All the acquired plasmids were validated by PCR and DNA sequencing.

### Protein Purification

Expression, purification, and quantification of the truncated AccB (AccB87) apo acceptor protein was performed as previously described^27^. The recombinant BirA1__LL_ and BirA2__LL_ proteins were purified from 1-L LB cultures grown at 37 °C to an OD_600_ of 0.8 and protein production was initiated by the addition of 0.5 mM IPTG for 4 h at 30 °C. Cell lysis and protein purification were performed using the previously described protocols^28^. The purified proteins were dialyzed overnight in storage buffer containing 50 mM Tris-HCl [pH 8.0], 150 mM KCl, 10% glycerol and 0.1 mM DTT, concentrated using Millipore concentrators, flash frozen, and stored at −80 °C. The purity of all the protein samples was judged by separation on 12% SDS-PAGE gels and staining with Coomassie brilliant blue.

### Liquid chromatography quadrupole time-of-flight mass spectrometry

A Waters Q-Tof API-US Quad-ToF mass spectrometer was used for the determination of the two *L. lactis* BirA orthologues (BirA1__LL_ & BirA2__LL_). The protein band of interest was removed from the gel and digested with Trypsin (G-Biosciences St. Louis, MO). Finally, the resultant peptides were loaded on a Waters Atlantis C-18 column (0.03 mm particle, 0.075 mm × 150 mm) and the acquired data were subjected for further analyses by the ms/ms.

### Bio-5′-AMP Synthesis Reactions

The assay for BirA-catalyzed *in vitro* protein biotinylation activity was performed as described previously^28^ with some modifications. Protein concentrations were determined using the extinction coefficients calculated from the protein sequence using the ExPASY Tools website. The assays contained 50 mM Tris-HCl (pH 8), 5 mM Tris-(2-carboxyethyl) phosphine, 5 mM MgCl_2_, 20 μM biotin, 5 μM ATP plus 16.5 nM [α-^32^P] ATP, 100 mM KCl and 2 μM BirA protein. Each of the reaction mixtures were incubated at 37 °C for 30 min. For each BirA protein tested, two identical tubes were used and at the end of the 30 min reaction AccB87 (50 μM) was added to one of each pair of tubes while the other tube was left untreated. The tubes were incubated for an additional 15 min at 37 °C. 1 μl of each reaction mixture was applied to a cellulose thin-layer chromatography plate of microcrystalline cellulose and the plates were developed in isobutyric acid-NH_4_OH-water (66:1:33)^29^. The thin-layer chromatograms were dried for 10 h, exposed to a phosphor-imaging plate and visualized using a Fujifilm FLA-3000 Phosphor Imager and Fujifilm Image Gauge software (version 3.4 for Mac OS).

### Mass spectrometry

MALDI TOF/TOF mass spectrometer was used to measure the level of BirA-catalyzed biotinylation of AccB87. Reactions containing 100 μM AccB-87, 3 μM BirA, and 100 μM biotin, 1 mM ATP, 10 mM MgCl_2_, 100 mM KCl, 5 mM tris-(2-carboxyethyl) phosphine in 50 mM Tris-HCl, (pH 8), at 37 °C, were incubated for 16 hours. Prior to the low-resolution matrix-assisted laser desorption/ionization analyses, the mixtures were dialyzed in 25 mM ammonium acetate and lyophilized to dryness. Data processing was performed using the FlexAnalysis 3.3 software package (Bruker Daltonics). Spectra were smoothed and a baseline correction was applied using the built-in features of the software package

### Electrophoretic mobility shift assays

Gel shift assays were performed to probe binding of BirA1_LL (and BirA2_LL) protein to the *bioY* promoters of *L. lactis* and *S. suis* 2 as previously described^26^,^30^,^31^. Two sets of DNA probes (*bioY*_LL and *bioY*_SS) were prepared by annealing two complementary oligonucleotides (*bioY*__LL_-F: 5′-CAA ATA ATA AAA TTA ACA GTT AAC CTA AAT TTG ATT TTA GGG TTA CTG TTT GAT ATG-3′; *bioY*__LL_-R: 5′-5′-CAT ATC AAA CAG TAA CCC TAA AAT CAA ATT TAG GTT AAC TGT TAA TTT TAT TAT TTG-3′). In the binding buffer (Roche), the purified BirA1_LL (and BirA2_LL) protein in a series of dilutions was mixed with the digoxigenin-labeled DNA probes (~0.2 pmol). If required, the biotinyl-5′-AMP ligand was added. The DNA/protein mixtures were separated on native 7% PAGE.

### Bioinformatics analyses

Both orthologues of BirA and the BirA-binding sites were subjected to multiple sequence alignments using the program of ClustalW2 (http://www.ebi.ac.uk/Tools/clustalw2/index.html), and the final outputs were given with the program ESPript 2.2 (http://espript.ibcp.fr/ESPript/cgi-bin/ESPript.cgi). The transcriptional start site was predicted using the Neutral Network Promoter Prediction server (http://www.fruitfly.org/seq_tools/promoter.html). Structural modelling was performed with the CPHmodels 3.2 Server (http://www.cbs.dtu.dk/services/CPHmodels) using appropriate structural templates: BirA_ec (PDB: 1HXD) for BirA1__LL_; BirA of *Aquifex aeolicus* (PDB: 3EFS) for BirA2__LL_; and the S component of ECF-type ABC transporter (PDB: 4DVE) is for two BioY orthologues of *L. lactis*.

### Transport of ^3^H-biotin

*L. lactis* was grown overnight in THB. A one ml sample was pelleted, washed three times in PBS, and used to inoculate minimal medium supplemented with either 1 nM or 1 μM of biotin. Samples were grown for 4.5 h, pelleted, and washed three times with PBS to remove external biotin. Pellets were then suspended in PBS plus 0.5% glucose for transport assays^6^ and quantification of total protein in each sample using the Bio-Rad Protein assay. For transport assays, samples were incubated with 0.5 μM ^3^H-biotin at 30 °C for 0.5, 1, 2, 5, 30, or 60 minutes. Reactions were halted via dilution in ice cold PBS and bacteria were collected on 0.45 μm filters. Filters were then mixed with scintillation fluid and DPM were counted using a Packard Scintillation counter.

## Additional Information

**How to cite this article**: Zhang, H. *et al*. Deciphering a unique biotin scavenging pathway with redundant genes in the probiotic bacterium *Lactococcus lactis. Sci. Rep.* **6**, 25680; doi: 10.1038/srep25680 (2016).

## Supplementary Material

Supplementary Information
