# Supplementary material for: Deciphering a unique biotin scavenging pathway with redundant genes in the probiotic bacterium Lactococcus lactis
Source: Sci Rep. 2016 May 10;6:25680. doi: 10.1038/srep25680 (PMC4861984; doi:10.1038/srep25680)
Supplement: Supplementary Information [file srep25680-s1.pdf]

Supplementary Information for

Deciphering a unique biotin scavenging pathway with redundant genes in the probiotic bacterium *Lactococcus lactis*

Huimin Zhang[1,4#], Qingjing Wang[1#], Derek J. Fisher[2], Mingzhu Cai[1], Vandana Chakravartty[3], Huiyan Ye[1], Ping Li[1], Jose O. Solbiati[4], Youjun Feng[1]*

[1]Department of Medical Microbiology and Parasitology, Zhejiang University School of Medicine, Hangzhou, Zhejiang 310058, PR China

[2]Department of Microbiology, Southern Illinois University, Carbondale, IL 62901, USA

[3]Department of Microbiology, University of Illinois, Urbana, IL 61801, USA

[4]Institute for Genomic Biology, University of Illinois, Urbana, Illinois 61801, USA

# These authors contributed equally to this work.

*To whom should be addressed: fengyj@zju.edu.cn (Y.F.)

Table S1 Comparative analyses of the genes involved in lipid metabolism of *L. lactis* and *S. suis* 2

Genes	*S. suis* 05ZYH33		*L. lactis* IL1403	
	Locus	GC %	Locus	GC %
Chromosome	/	41.1	/	35.3
*birA*1	SSU05_1625	44.0	L0191	34.2
*birA*2	/	/	L0192	34.4
*bioY*1	SSU05_0509	40.6	L24031	35.4
*bioY*2	/	/	L1011	37.5
pycA			L63652	38.1
accA	SSU05_1796	52.4	L0190	38.6
accB	SSU05_1801	53.1	L0187	38.7
accD1	SSU05_1797	51.3	L0180	37.8
accD2	SSU05_1798	42.9	/	/
accC	SSU05_1799	49.2	L0189	39.0
fadA	/	/	L25946	36.3
fadB	/	/	/	/
fadL	/	/	/	/
fadD	/	/	L54546	37.3
fadE	/	/	/	/
fabF	SSU05_1802	46.2	L0186	41.0
*fabG*1	SSU05_1803	43.4	L0185	36.9
*fabG*2	/	/	L27694	35.6
*fabG*3	/	/	L1530	41.7
*fabZ*1	SSU05_1800	45.5	L160425	34.9
fabZ	/	/	L0188	37.7
fabI	/	/	L161132	38.9
fabH	SSU05_1807	46.1	L0182	40.4
fabD	SSU05_1804	47.6	L0184	37.0
fabK	SSU05_1805	43.8	/	/
acpP	SSU05_1806	37.4	L0183	36.0
acpD			L115551	36.1
acpH	/	/	L186107	36.5
fabT	SSU05_1808	39.1	L180805	35.3
fabM	SSU05_1809	43.2	/	/
acpS	SSU05_1813	43.4	L61355	36.7
lplA	SSU05_1836	43.5	L64373	37.9
